# Dual-endoscopy detection for an esophageal-jejunal anastomotic fistula

**DOI:** 10.1055/a-2107-2540

**Published:** 2023-07-11

**Authors:** Zhengying Yang, Ying Bi, Jinfeng Ren, Xihui Yuan, Neng Wang, Tianyu Liu

**Affiliations:** 1 Fourth Department, Digestive Disease Center, Suining Central Hospital, Sichuan, China; 2 School of Medical and Life Sciences, Chengdu University of Traditional Chinese Medicine, Sichuan, China; 3 Department of Digestive Endoscopy Center, Suining Central Hospital, Sichuan, China

An anastomotic fistula is a severe complication of post-gastrectomy. In the past, re-surgery has been the most common method to address this complication 1 . However, it may bring many subsequent complications 2 . With the development of endoscopic techniques and related accessories, endoscopy is gradually able to address more post-surgical complications. Here, we report a case of dual-endoscopy detection and suture of an esophageal-jejunal anastomosis fistula.

A 72-year-old man was admitted to the hospital complaining of food leakage from the abdominal drainage tube over the past 4 months. The upper gastrointestinal contrast revealed partial contrast medium flowing out of the drainage tube in the anastomotic site ( Fig. 1 ). The gastric endoscopy showed a drainage tube inserted into the intestinal lumen from the anastomosis orifice ( Fig. 2 a ). Endoscopic treatment was performed after the patient’s consent. First the drainage tube was removed. The dual-endoscopy detection combined with a superfine gastroscope (Olympus GIF-HQ290; Olympus, Tokyo, Japan) and conventional transoral gastroscope (Olympus GIF-Q260 J) was performed simultaneously by two operators ( Video 1 ). The superfine gastroscope was inserted through the sinus tract and docked with the conventional gastroscope. The anastomosis fistula was then sutured with nylon rope and metallic clips in a purse-string manner under the conventional gastroscope. Because the docked dual-endoscopy detection showed the sinus tract was continuous with no infection in the anastomosis and sinus tract, the lateral serous membrane was not sutured. The follow-up gastroscopy 2 months later showed complete healing of the anastomosis ( Fig. 2 b ) and fistula tract.

**Fig. 1:**
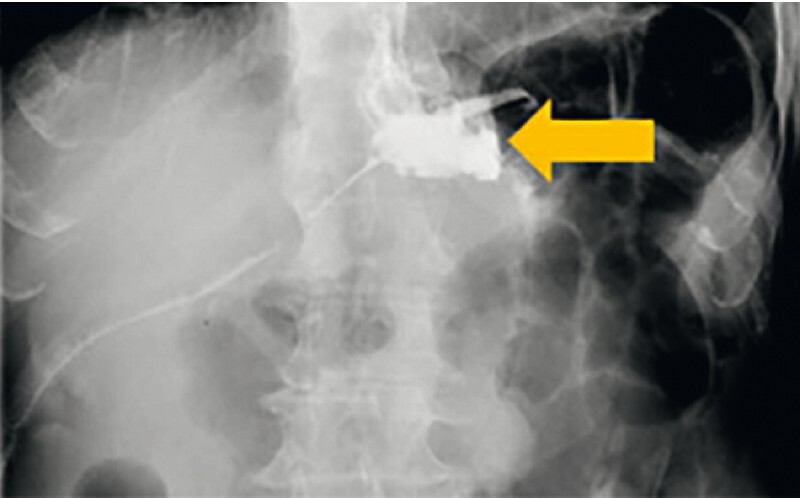
Upper gastrointestinal contrast showing partial contrast medium flowing out of the drainage tube in the anastomotic site.

**Fig. 2 a:**
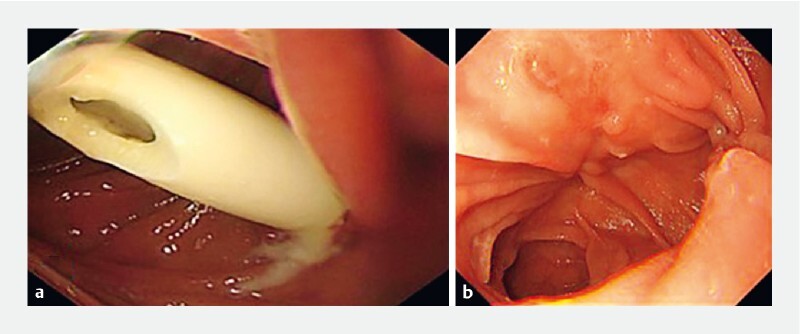
Gastroscope showing a drainage tube inserted into the intestinal lumen from the inferior wall of the anastomosis orifice. **b** Follow-up gastroscopy 2 months later showing complete healing of anastomosis.

**Video 1** Dual-endoscopy detection combined with a superfine gastroscope and conventional transoral gastroscope was performed simultaneously.


Dual-endoscopy detection could be applied to determine the therapeutic plan of patients with post-surgical anastomotic fistula and enterocutaneous fistula by detecting whether the sinus tract is continuous, infected, purulent, etc. After confirmation of the leak in the sinus tract and no infection, the suture of the lateral serous membrane is not needed and the sinus tract can be closed without additional processing. Long-term follow-up should be planned for further assessment.

Endoscopy_UCTN_Code_TTT_1AO_2AB

## Notes

Endoscopy E-Videos

https://eref.thieme.de/e-videos
*E-Videos* is an open access online section of the journal *Endoscopy* , reporting on interesting cases and new techniques in gastroenterological endoscopy. All papers include a high-quality video and are published with a Creative Commons CC-BY license. Endoscopy E-Videos qualify for HINARI discounts and waivers and eligibility is automatically checked during the submission process. We grant 100% waivers to articles whose corresponding authors are based in Group A countries and 50% waivers to those who are based in Group B countries as classified by Research4Life (see: https://www.research4life.org/access/eligibility/ ). This section has its own submission website at https://mc.manuscriptcentral.com/e-videos
